# Juvenile Systemic Lupus Erythematosus Presenting with Esophagitis and Severe Oral Mucositis

**DOI:** 10.1155/2021/5868655

**Published:** 2021-06-02

**Authors:** Emily Schildt, Kristen L Sessions, Deirdre De Ranieri

**Affiliations:** ^1^Ann & Robert H. Lurie Children's Hospital of Chicago, Chicago, IL, USA; ^2^Ann & Robert H. Lurie Children's Hospital of Chicago, Department of Rheumatology, Chicago, IL, USA

## Abstract

We present a case of a previously healthy adolescent female who developed severe oral mucositis and acute esophagitis as her presenting symptoms of juvenile systemic lupus erythematosus. Mucositis involving the lips is infrequently reported in systemic lupus erythematosus, and to our knowledge, this is the first reported case of acute, non-infectious esophagitis as a presenting symptom in a pediatric systemic lupus erythematosus patient.

## 1. Introduction

Systemic lupus erythematosus (SLE) is a complex, multisystem autoimmune disease that can present with a variety of symptoms in both the adult and pediatric populations. Using data from nationwide studies that looked at Medicaid-enrolled children, the incidence of juvenile SLE (jSLE) is approximately 2.2 per 100,000/year and the prevalence is approximately 9.7 per 100,000 [1]. SLE is rare before the age of 5 and increasingly more common after the first decade of life [2]. The kidneys, heart, lung, and brain are the most commonly affected organs, but virtually any organ can be involved. Oral and nasal ulcerations are part of the 2012 Systemic Lupus Collaborating Clinics (SLICC) diagnostic criteria, and oral ulcerations are also on the EULAR/ACR 2019 diagnostic criteria, with oral lesions occurring in up to 54% of patients with SLE [3–5]. These are classically painless ulcers on the hard palate; lip involvement, often referred to as lupus cheilitis, is less frequently reported in the literature, but it is a known manifestation of SLE and jSLE [6–8]. However, to our knowledge, there are no reported cases of acute, non-infectious esophagitis as the presenting symptoms of SLE in a pediatric patient.

## 2. Case Presentation

A 13-year-old girl presented with a one-week history of lip swelling, ulceration, and bleeding, complicated by moderate dehydration. Review of systems was notable for odynophagia, fatigue, and one day of fever. Chart review revealed an 8.5 kg weight loss over the past 3 months. She was previously healthy and not on any medications.

**Figure 1:**
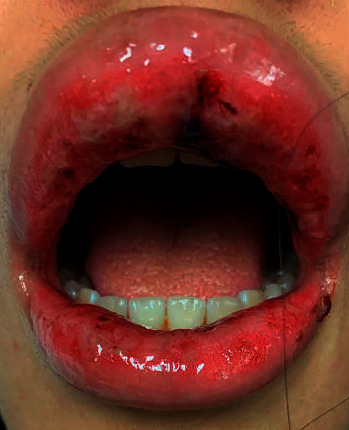
Ulcerated and edematous upper and lower lips at the time of presentation.

**Figure 2:**
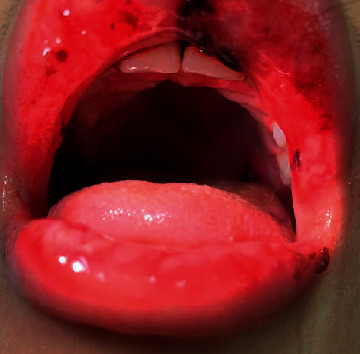
Oral ulcer on the left posterior palate at the time of presentation.

Her exam was significant for diffusely edematous and erythematous upper and lower lips with cracking, blistering, and evidence of prior bleeding (Figure 1). She had erythema and dried blood visible on her upper and lower gingivae without discrete lesions. She additionally had a 5 mm dark red ulceration on her posterior palate (Figure 2). No aphthous ulcerations or vesicular lesions were present on her oral mucosa. She had no nasal, labial, or perianal mucosal changes and no skin rashes. Her joints were unremarkable without swelling, pain, or stiffness.

Initial laboratory studies were significant for leukopenia with prominent lymphopenia, normocytic anemia, borderline thrombocytopenia, elevated ESR, elevated BUN and creatinine, hematuria, and proteinuria. CRP was normal (Table 1).

Multiple subspecialties were consulted for workup, including rheumatology, oncology, gastroenterology, and nephrology. She underwent a CT scan of her chest/abdomen/pelvis as well as a bone marrow biopsy, a renal biopsy, an upper endoscopy with biopsies, and a lip biopsy for further evaluation.

**Figure 3:**
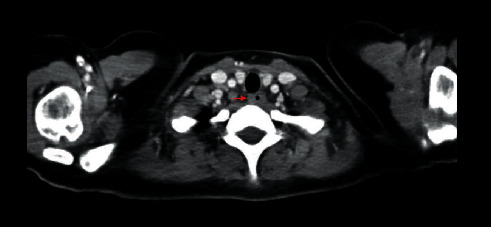
CT chest/abdomen/pelvis demonstrating diffuse, circumferential esophageal thickening as denoted by red arrow.

CT scan revealed circumferential thickening of her esophagus (Figure 3) and diffuse lymphadenopathy. Bone marrow biopsy and peripheral blood analysis were not consistent with malignancy, with slight hypocellularity but normal cell lines.

**Figure 4:**
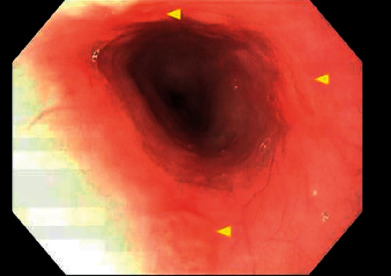
Upper endoscopy with gross visualization of shallow ulcerations in the esophageal mucosa.

Upper endoscopy with biopsies was performed and revealed shallow ulcerations throughout her esophagus (Figure 4). Histology was significant for acute esophagitis, with few neutrophils and prominent epithelial reactive changes in the squamous mucosa. The mucosa in her duodenum and stomach appeared normal although there was evidence of chronic inactive gastritis on biopsy. Lip biopsy pathology resulted as interface dermatitis with lymphocytic infiltrate and focal vacuolar changes of the basal cells with eosinophils, and notable ulcerations with apoptotic epithelial cells, consistent with cutaneous involvement from lupus [9].

Infectious workup was negative, including HIV, mycoplasma, HSV, and EBV serum serologies. Serum CMV IgM was positive, but with negative serum CMV PCR, negative CMV staining of esophageal biopsy, and no characteristic findings on biopsy concerning for CMV. Esophageal biopsy was also negative for HSV staining, and fungal elements and swab of lip lesions were negative for HSV.

Rheumatologic workup was notable for highly elevated ANA and anti-dsDNA by Crithidia IFA (both >2560), hypocomplementemia (low C3, C4, and CH50), positive Coombs, and nephrotic range proteinuria (Table 2). Kidney biopsy demonstrated Class IV lupus nephritis. She was then diagnosed with jSLE.

During her clinical course, she required central line placement and initiation of TPN given her inability to eat and drink due to severe odynophagia. Once the diagnosis of SLE was confirmed, she was treated with daily pulse-dose IV methylprednisolone (1g) for 5 days, with marked improvement in the appearance of her lip swelling and mucositis. She was subsequently transitioned to oral prednisone. Given the presence of Class IV lupus nephritis, induction therapy with mycophenolate mofetil was initiated, and hydroxychloroquine was added as an additional immunomodulator.

## 3. Discussion

The initial workup of our patient was broad and involved the coordination of multiple subspecialties as well as invasive procedures. At the time of presentation, her symptoms and exam findings were thought to be consistent with a viral etiology such as mycoplasma-induced mucositis and rash (MIRM), HSV, CMV, EBV, or HIV, with an additional consideration for possible fungal etiologies causing esophagitis. However, her initial labs and history of weight loss were concerning for a more systemic inflammatory process, which broadened the differential diagnosis to include rheumatologic and oncologic etiologies such as SLE, vasculitis, leukemia, lymphoma, or Castleman disease with paraneoplastic pemphigus. After an extensive workup including imaging studies, laboratory evaluation, and biopsies of multiple sites, diagnosis of jSLE was made, with the presence of cytopenias, hypocomplementemia, abnormal serologies, and renal disease fulfilling the criteria.

Juvenile SLE is a multisystem autoimmune disease with a wide range of phenotypes. Mucosal involvement, most commonly as hard palate ulcers, is common. However, this is the first reported case of esophagitis as a presenting symptom of jSLE. There are several studies and cases describing the presence of dysphagia associated with esophageal dysmotility in many connective tissue conditions, but acute esophagitis is very rarely associated with SLE [10]. When it has been described, it has been in association with infectious etiologies, likely secondary to an immunocompromised state [11, 12]. However, no infectious etiology was found in our patient. Additionally, two case reports reported sloughing esophageal mucosa in association with bullous SLE in adults, although this is a unique histopathology and was not consistent with clinical or pathologic findings in our patient [13, 14].

Oral ulcerations are a commonly described finding in SLE, as it is one of the diagnostic criteria, and may be more common in jSLE than adult SLE [15]. The most frequent manifestations of oral lesions in SLE are painless palatal ulcers and aphthous ulcers on the buccal mucosa [6, 7]. Lip-specific findings have been reported but are much less frequent than the classic SLE oral manifestations [8]. Of the described lip findings in SLE, many are associated with discoid lupus. There are rare reports of patients with SLE presenting with angioedema or cheilitis with crusting [16–18].

In conclusion, it is important to recognize mucosal ulceration as a manifestation of jSLE. Our patient's initial presentation with ulceration of the lips and esophagus was initially thought to be virally mediated but later found to be secondary to evolving jSLE. To our knowledge, this is the first case of jSLE presenting as acute non-infectious esophagitis. This case illustrates the importance of maintaining a broad differential diagnosis when approaching a patient with unusual symptoms and laboratory findings and considering jSLE as an etiology for abnormal mucosal findings in a child.

## Figures and Tables

**Table 1:** Initial laboratory studies at the time of presentation (abnormal findings are indicated in bold).

Labs	Ref ranges	Actual values
White blood cells (thou/uL)	4.5–13.5	**2.59**
Hemoglobin (g/dL)	12.0–16.0	**9.6**
Hematocrit (%)	36–46	**29.8**
MCV (fL)	78.0–98.0	85.9
Platelets (thou/uL)	150–450	155
Absolute lymphocyte count (thou/uL)	1.2–7.83	**0.340**
Absolute neutrophil count (thou/uL)	1.7–9.715	2.119
Reticulocytes (%)	0.5–2.5	0.7
Ferritin (ng/mL)	11–320	**839**
Albumin (g/dL)	3.6–4.7	**2.9**
Creatinine (mg/dL)	0.25–0.94	**1.16**
BUN (mg/dL)	7–18	**36**
UA		Large occult blood, >500 protein
Urine protein/creatinine	<0.2	**2.9**

**Table 2:** Rheumatologic workup (abnormal findings are indicated in bold).

Labs	Ref ranges	Actual values
ANA (titer)	<40	**>2560**
Anti-dsDNA, Crithidia IFA^*∗*^ (titer)	<10	**>2560**
Anti-SS-A/Ro (EIA)	0–19.99	**33.7**
Anti-SS-B/La (EIA)	0–19.99	4.3
C3 (mg/dL)	86–184	**12.0**
C4 (mg/dL)	20–59	**4.34**
Total hemolytic complement (CAE)	70–200	**6.7**
Coombs, direct and indirect	Negative	**Positive Anti-IgG**
Anti-Cardiolipin IgG (GPL)	0–14.9	**16.1**
Anti-Cardiolipin IgM (MPL)	0–12.4	**18.4**
Anti-Beta-2 Glycoprotein IgG (SGU)	0–19.9	7.5
Anti-Beta-2 Glycoprotein IgM (SGU)	0–19.9	**28.0**
PTT-LA screen (seconds)	≤40	34
dRVVT screen (seconds)	≤45	35
Anti-Smith Abs (EIA)	0–19.9	14.1
U1RNP (EIA)	0–19.9	**21.8**
ESR (mm/hr)	0–20	**93**
CRP (mg/dL)	0–0.8	<0.3

^*∗*^Anti-dsDNA Crithidia testing manufactured by Bio-Rad Laboratories.

## Data Availability

Requests for access to the full data of this case report can be made to the corresponding author at eschildt@luriechildrens.org.
